# Reduction of Posttraumatic Stress Disorder (PTSD) Symptoms in PTSD and Major Depressive Disorder Comorbidity After Acute Hypoglycemia—A Case Report

**DOI:** 10.3389/fpsyt.2019.00530

**Published:** 2019-07-26

**Authors:** Tomasz Pawlowski, Jacek Daroszewski, Agnieszka Czerwinska, Joanna Rymaszewska

**Affiliations:** ^1^Division of Psychotherapy and Psychosomatic Medicine, Department of Psychiatry, Wroclaw Medical University, Wroclaw, Poland; ^2^Department of Endocrinology, Diabetes and Isotope Therapy, Wroclaw Medical University, Wroclaw, Poland; ^3^Department of Psychiatry, Wroclaw Medical University, Wroclaw, Poland

**Keywords:** psychoneuroendocrinology, posttraumatic stress disorder, major depressive disorder, acute hypoglycemia, insulin tolerance test

## Abstract

**Background:** Approximately half of all patients with posttraumatic stress disorder (PTSD) also suffer from major depressive disorder (MDD). This co-occurrence might lead to an impairment of cognitive functions, worse response to antidepressant medications, and an increased risk of suicide in comparison to patients with PTSD alone. Prognosis for people with PTSD and MDD co-occurrence is poorer than for either one alone; therefore, researchers look for novel, effective treatments.

**Case Presentation:** A patient with MDD with the co-occurrence of PTSD was admitted to the Department of Endocrinology with suspicion of adrenal insufficiency. In order to assess the adrenocorticotropin/cortisol axis, a standard insulin tolerance test was performed. After inducing a hypoglycemic episode with intravenous short-acting insulin, PTSD symptoms were reduced. To the best of our knowledge, this is the first report on the reduction of PTSD symptoms after performing an insulin tolerance test.

**Conclusion:** Reduction of PTSD symptoms in PTSD and MDD comorbidity has been noticed after a hypoglycemic episode. This demonstrates the mutual dependencies between the endocrine and nervous systems, covered extensively by psychoneuroendocrinology.

## Background

Meta-analysis data indicate that in as many as 52% patients with a posttraumatic stress disorder (PTSD), major depressive disorder (MDD) co-occurs, which might lead to an impairment of cognitive functions, worse response to antidepressant medications, and an increased risk of suicide in comparison to patients with PTSD alone (1). Neuroimaging studies reveal differences between people with PTSD and MDD and those with PTSD alone (2–4). In the PTSD/MDD group, medial prefrontal cortex and amygdala activation by threatening stimuli are lower than in the PTSD group. People with PTSD/MDD also exhibit a lower functional connectivity between the insula and hippocampus when compared to those with PTSD alone. Thus, it can be hypothesized that PTSD and MDD comorbidity can even represent a subtype of PTSD (5).

Food and Drug Administration (FDA)–approved pharmacological treatment for PTSD produces a response rate of approximately 60% (6). Prognosis for people with PTSD and MDD co-occurrence is poorer than for either one alone. Dropout rates in the PTSD/MDD group are more common; therefore, researchers look for novel, effective treatments. For example, the results of the Yehuda et al. study reveal that hydrocortisone augmentation during prolonged exposure therapy may result in a greater retention in the treatment group, thereby improving PTSD symptom response (7).

Here we describe a case of a female patient with MDD with the co-occurrence of PTSD diagnosed in the Department of Endocrinology, where she was admitted with suspicion of adrenal insufficiency. The insulin tolerance test (ITT) was performed to diagnose adrenal insufficiency. After inducing a hypoglycemic episode with intravenous short-acting insulin, PTSD symptoms were reduced.

## Case Presentation

The patient was a 36-year-old woman, married, with no children; having completed a higher education, she is a lawyer by profession. In October 2013, the patient sought psychiatric help in an outpatient clinic due to depressed mood, decreased interest, insomnia, feelings of worthlessness, and significant weight change (−10%). MDD, 296.22 [*Diagnostic and Statistical Manual of Mental Disorders, Fourth Edition, Text Revision* (*DSM-IV-TR*)] was diagnosed, and the patient received escitalopram 20 mg/d, with no improvement after 5 weeks’ treatment. Thus, the treatment changed to paroxetine 60 mg/d and trazodone 150 mg/d, which resulted in a marked improvement within 6 weeks (difficulties in starting activities remained).

In January 2014, the patient was diagnosed with Hashimoto autoimmune thyroiditis. Introduction of levothyroxine 75 mg/d was temporally connected to remission of depressive symptoms. The diagnosis was updated to mood disorder due to hypothyroidism with major depressive-like episode, 293.83 (*DSM-IV-TR*). In March 2015, psychiatric treatment was concluded.

In May 2015, the patient became pregnant. Ultrasound examination revealed multiple birth defects of the fetus, which led to its death in week 16 of the pregnancy. The patient was hospitalized in the Department of Gynaecology and Obstetrics, where she gave birth to a dead fetus. During the event, she experienced intense fear and helplessness. Following the incident, the patient sought psychiatric help, again due to symptoms she had experienced earlier and which had returned, that is, depressed mood, decreased interest, insomnia, and feelings of worthlessness. Additional symptoms of PTSD, unreported previously, ensued: recurrent distressing dreams of the event, the sensation that the traumatic event was recurring, and the inability to recall an important aspect of the trauma (the patient did not remember a part of her stay at the gynecological ward). Attempts at broaching the subject of losing her child were met with defiance and emotional withdrawal. Her general state was characterized by an increased arousal typical of PTSD cases (irritability, outbursts of anger, difficulty concentrating, hypervigilance). The patient no longer managed to function normally in social contexts (workplace, home). MDD, 296.22, was diagnosed with the co-occurrence of acute PTSD, 309.81. Given that selective serotonin reuptake inhibitor (SSRI) medications such as sertraline and paroxetine are approved by the FDA for the treatment of PTSD and with her previous treatment response, we decided to start with paroxetine 20 mg/d and trazodone 75 mg/d. However, no marked improvement was noted following 5 weeks of treatment. Paroxetine was increased up to 60 mg/d and trazodone up to 150 mg/d, the same dose prescribed previously, which has resulted in marked improvement of her MDD.

Due to a massive sleep dysfunction consisting in troubles with falling asleep, frequent waking periods during the night, and a very high level of irritability, as well as multiple outbursts of anger and anxiety, it was necessary to increase the trazodone dose to 200 mg/d and to introduce diazepam in the dose of 5 mg/tds. Moreover, the patient’s somatic health condition was deteriorating rapidly. The patient lost weight [−10% within a month; body mass index (BMI) = 19 kg/m^2^]; she complained of general weakness, dizziness and fainting, as well as of episodes of hyperventilation and paresthesia. Physical examination revealed dehydration and low blood pressure (100/60 mmHg), which plummeted even further when standing (orthostatic hypotension). Assessments of both morning adrenocorticotropin (ACTH) and morning cortisol levels in the blood performed on an outpatient basis revealed slightly decreased results (ACTH = 6.00 pg/ml; cortisol = 4.90 ng/ml). Therefore, in April 2016, the patient was admitted to the Department of Endocrinology with suspicion of adrenal insufficiency.

During the patient’s stay at the ward, examination revealed correct circadian rhythm in serum cortisol (6:00, 5.60 μg/dl; 8:00, 11.30 μg/dl; 20:00, 4.40 μg/dl; 24:00, 4.20 μg/dl), as well as correct levels of the hormone in a 24-h sample of urine: 28.2 μg/24. Also, the ACTH concentration in the daily profile of the serum was correct (6:00, 6.12 pg/ml; 8:00, 10.20 pg/ml; 20:00, <5.40 pg/ml; 24:00, 7.07 pg/ml). Thyrotropin concentration (TSH) with both free thyroxine (fT4) and free triiodothyronine (fT3) were within normal range, with elevated anti-thyroid peroxidase (TPO) antibodies, 253 IU/ml (reference range < 35 IU/ml).

In order to assess the ACTH/cortisol axis for detecting secondary adrenal insufficiency, a standard ITT was performed. Symptomatic hypoglycemia, with blood glucose values below 40 mg/dl, was required to evoke a reliable central stress response with the activation of the hypothalamic–pituitary–adrenal (HPA) axis. Intravenous insulin was administered (0.1 units/kg; 6j aspart insulin). Symptomatic hypoglycemia, with blood glucose values of 29 mg/dl within 30 min of the test, was achieved. The patient experienced mild palpitations, massive hot flushes, and sweating, which disappeared within several minutes of the procedure. The results of the test excluded any abnormalities of the ACTH/cortisol and growth hormone secretion (**Table 1**).

**Table 1:** Insulin tolerance test: documented hypoglycemia; subsequent rise of adrenocorticotropin (ACTH), cortisol, and growth hormone (GH) concentrations.

Parameter/time	0 min	15 min	30 min	45 min	60 min	90 min	120 min
Glucose (mg/dl)	88	47	29	36	42	59	75
GH (ng/ml)	0.27	0.54	7.92	17.0	22.9	21.8	9.39
ACTH (pg/ml)	7.04	5.98	12.80	116.0	111.0	50.3	24.1
Cortisol (µg/dl)	5.0	6.9	6.5	12.8	14.9	15.5	16.4

Four days after hospitalization, the patient had a consultation with her psychiatrist (the first author of this manuscript). During the examination, a marked improvement of the patient’s mental state was noted. Symptoms of PTSD were reduced: increased arousal diminished, sleep was normalized, and bouts of anxiety became much less frequent and less severe. The patient was able to recall an important aspect of the trauma, and recurrent distressing dreams of the event disappeared. MDD symptoms persisted in the clinical picture, including depressed mood, decreased interest, and feelings of worthlessness. Such results enabled the psychiatrist to take the patient off diazepam (in the course of 10 days).

Over the following month, MDD symptoms gradually became less intense; consequently, trazodone was reduced from 200 to 100 mg/d, and paroxetine from 60 to 40 mg/d. In July 2017, 14 months after the ITT, pharmacological treatment was concluded, and presently, the patient remains in remission and is planning a pregnancy.

## Discussion

To the best of our knowledge, this is the first report on the reduction of PTSD symptoms after hypoglycemia induced during a standard ITT. The effects of insulin on the central nervous system were first reported by the Austrian-American psychiatrist Manfred Sakel, who applied insulin so as to decrease anxiety and agitation in patients on a withdrawal course from opioids. This was further expanded into insulin shock therapy, originally used to treat schizophrenia and later also depressive disorders (8). The clinical mechanism of what seemingly rendered the procedure successful has not been scientifically proven.

In the present case report, the improvement of the patient’s mental condition might stem both from systemic functions of insulin, which cause hypoglycemia, and from the effect of insulin on the central nervous system. Florez et al. describe the impact of hypoglycemia on the intrinsic CA (cornu ammonis region) 3 rhythm of the hippocampus (9). A drop in the glucose perfusate concentration produced seizure-like events (SLEs) originating in the CA3 region and spreading into the CA1 region. Prior to the onset of the SLE in the CA3 area, decreased gamma-aminobutyric acid (GABA)–correlated baseline sharp wave activity was observed, simultaneously with increased large-amplitude sharp wave activity from synchronous pyramidal cell firing. Hypoglycemia induces the release of aspartate and glutamate, and transiently GABA, which moves the balance of the circuitry towards excitation. Significantly lower GABA levels have been detected in PTSD (10), which seems to explain why benzodiazepines remain popular forms of treatment in PTSD—they enhance the effect of GABA.

Patients with PTSD/MDD resemble those with PTSD alone in their HPA axis response (11). In patients with PTSD/MDD, peripheral cortisol levels are generally lower, with enhanced glucocorticoid receptor (GR) sensitivity as inferred from results of the dexamethasone suppression test (DST). Patients with PTSD have an exaggerated cortisol suppression response to dexamethasone (DEX), which indicates that the negative-feedback system of the HPA axis is overly sensitive. In healthy subjects, the administration of intranasal insulin prior to a psychosocial stressor was found to diminish saliva and plasma cortisol without affecting heart rate or blood pressure stress reactivity, suggesting that insulin blunts the responsiveness of the stress-induced HPA axis (12).

## Conclusions

In this case report, it has been observed that induction of a hypoglycemic episode with intravenous short-acting insulin was temporally connected to improvement of PTSD symptoms in PTSD and MDD comorbidity. The present case study demonstrates the mutual dependencies between the endocrine and nervous systems, covered extensively by psychoneuroendocrinology.

## Ethics Statement

Written informed consent was obtained from the patient for the publication of this case report.

## Author Contributions

Specific author contributions: study design (TP, AC), data collection (TP, JD), analysis (TP, JD, JR, AC), manuscript writing (TP, JD, JR, CA).

## Funding

Funding has been provided through grant ST.C230.18.014 from Wroclaw Medical University.

## Conflict of Interest Statement

The authors declare that the research was conducted in the absence of any commercial or financial relationships that could be construed as a potential conflict of interest.
